# Elevated preoperative heart rate associated with increased risk of cardiopulmonary complications after resection for lung cancer

**DOI:** 10.1186/s12871-018-0558-9

**Published:** 2018-07-25

**Authors:** Danxia Fu, Chaoshuang Wu, Xiaoyu Li, Junping Chen

**Affiliations:** Department of anesthesiology, Ningbo NO.2 hospital, NO.41 Xibei Street, Ningbo City, 315000 Zhejiang Province China

**Keywords:** Resting heart rate, Cardiopulmonary complications, Risk assessment, Lung cancer

## Abstract

**Background:**

The study aimed to assess whether preoperative resting heart rate could be a risk factor for cardiopulmonary complications (CPCs) after lung cancer resection.

**Methods:**

Eligible consecutive patients who underwent resection surgery for non-small cell lung cancer (NSCLC) at Ningbo NO.2 Hospital between May, 2010 and July, 2015 were included. The demographic, clinical characteristics and laboratory parameters were compared in patients with or without CPCs within postoperative 30 days. The multivariate logistic regression analysis was used to analyze the association between CPCs and risk factors. Receiver operating characteristic (ROC) curve analysis was utilized for the predictive role of preoperative resting heart rate for CPCs.

**Results:**

One hundred eighty participants were enrolled into the final analysis and 42 of them had an established diagnosis of CPCs within postoperative 30 days. Elevated preoperative resting heart rate was an independent risk factor for postoperative CPCs (OR: 4.48, 95% CI: 1.17–18.42, *P* = 0.021) by the multivariate logistic regression analysis. ROC curve analysis indicated elevated resting heart rate as a predictor for CPCs with a cut-off value of 86 beats/min (AUC: 0.813, specificity: 80.95%, sensitivity: 72.46%, *P* < 0.001).

**Conclusions:**

Elevated preoperative resting heart rate was associated with an increased risk of postoperative CPCs in patients after resection for lung cancer.

## Background

Lung resection is the standard therapeutic strategy for early-stage lung cancer management and it provides the best chance of cure [1]. Lung resection can always result in lung function impairment, a higher incidence of postoperative complications and mortality in comparison with some other surgical disciplines [2]. Previous data has reported a mortality rate for lung resection surgery as high as 6.7% and cardiopulmonary complications (CPCs) are major causes for postoperative mortality and morbidity [3]. Therefore, appropriate preoperative management and valid CPCs prediction are of great importance in minimizing the morbidity or mortality. Pulmonary function and cardiopulmonary fitness measurement have been suggested for patient risk stratification [4, 5]. Some other parameters such as gender, body mass index (BMI), renal dysfunction, etc. are also suggested as risk factors for CPCs [6]. A close association has been demonstrated between resting heart rate and an elevated incidence of cardiovascular mortality both in patients with cardiac diseases and general population [7]. Previous data have indicated the predictive value of increased heart rate for unfavorable outcomes in those patients with an acute coronary syndrome or heart failure [8]. Recent studies have revealed that elevated preoperative resting heart rate (> 87 beats/min) significantly correlates with postoperative myocardial impairment and mortality in patients undergoing non-cardiac surgery [9].

To reduce selection bias, only one type of surgery (lung cancer) was chosen in this study. In comparison with other non-cardiac surgery, thoracic surgery shows a higher morbidity rate of CPCs. Due to the high morbidity rate and mortality, establishing an association between heart rate and CPCs is of great importance for risk stratification. However, few data has specifically explored the relationship between heart rate and postoperative CPCs in patients with lung cancer. The current study aimed to assess potential risk factors for the CPCs after resection for lung cancer.

## Methods

### Patients

This study was approved by the Medical Institutional Ethics Committee of Zhejiang province. We prospectively reviewed eligible consecutive patients who underwent resection surgery for non-small cell lung cancer (NSCLC) at Ningbo NO.2 Hospital between May, 2010 and July, 2015. Patients enrolled were required to provide written informed consent before the study.

### Data collection

Demographics data were collected preoperatively set from patients and their medical records including age, gender, body mass index (BMI), smoking habits, American Society of Anesthesiologists (ASA) physical status, preoperative pre-existing cardiovascular medications, etc. The preoperative comorbidities including diabetes, hypertension, chronic obstructive pulmonary disease (COPD), coronary artery disease (CAD), chronic kidney disease (CKD), prior myocardial infarction and atrial fibrillation were also recorded in details.

All participants enrolled were required to undergo electrocardiograms, pulmonary function tests and blood gas analysis before the operation. Preoperative evaluation of Revised Cardiac Risk Index (RCRI) and cardiovascular comorbidities were according to the updated American College of Cardiology (ACC)/American Heart Association (AHA) Task Force guidelines [10]. Those patients with cardiac risks or diseases (cardiac surgery history, ischemic heart disease, etc.) underwent full physical evaluation by an experienced cardiologist. If necessary, a further echocardiographic test or invasive evaluation examinations was conducted to assess their cardiac status deeply. The clinical data including tumor location, clinical stage, nodal stage and types of surgery were also documented according to the observations during operation and postoperative pathological results. Baseline resting heart rate was measured using a digital monitor after patients have sat and rested for at least 5 min. Readings were taken for three times and the mean value was recorded.

The primary end point of this study was CPCs within postoperative 30 days.

### Definition of CPCs

The definition of postoperative CPCs was according to the descriptions by Duc et al. [11], including pleural effusion requiring drainage, mechanical ventilation within postoperative 48 h, atelectasis, acute respiratory distress syndrome (ARDS), respiratory failure, pneumonia, pneumothorax, pulmonary embolism, empyema, ventricular or atrial arrhythmias, myocardial infarction.

### Laboratory tests

Fasting blood samples were obtained in the morning on 1 day prior to the operation from all participants. The concentrations of cardiac troponin T (cTnT) and inflammatory cytokines including C-reactive protein (CRP) and tumor necrosis factor-α (TNF-α) were measured using enzyme-linked immunesorbent assays (ELISA) according to the manufacturers’ instructions (R&D Systems, Minneapolis, MN, USA). The expressions of hemoglobin, albumin, creatinine and urea were also measured from the blood samples.

### Statistical analysis

SPSS 19.0 (SPSS, Inc.) and Graphpad 5.0 (Instat, San Diego, CA) were used for statistical analysis in our study. A sample size calculation was performed before the study and at least 138 patients would be required using a 5% significance level and 80% power. We used the estimated incidence of CPCs as a basis for the sample size estimation according to the reference values reported and our previous clinical experience. Data are given as number (n) with percentage (%), or mean ± standard error (SD). Chi-squared test or Fisher’s exact test was used for the statistical analysis of categorical variables. Numeric variables were analyzed by unpaired t test (for normal distribution) or Mann Whitney test (for non-normal distribution). We used multivariate logistic regression analysis to analyze for association between CPCs and risk factors. In the logistic regression model, the categorical data (including CRP, TNF-α, etc.) were grouped into high group vs low group with the median level as the cut-off value. Receiver operating characteristic (ROC) curve analysis was utilized for the predictive role of resting heart rate for CPCs. All statistical tests were bilateral probability and a *P* value of < 0.05 was accepted as statistically significant.

## Results

### Patient characteristics

Two hundred three participants were recruited into this study between May, 2010 and July, 2015. After excluding 23 patients with missing data or refusal of cooperation, the remained 180 NSCLC patients were enrolled into the final analysis. The mean age of participants was 62.6 years and 88 (48.9%) were male patients. Of all the 180 NSCLC patients, 106 (58.9%) were squamous cell carcinoma, 65 (36.1%) were adenocarcinoma and 9 (5.0%) were mixed. The detailed patient characteristics are presented in Tables 1 and 42 of the 180 (23.3%) had an established diagnosis of CPCs within postoperative 30 days. Pleural effusion (15/180, 8.3%) and arrhythmias (11/180, 6.1%) were the two most common complications in this series, which is shown in Table 1. There were no statistical differences between patients in CPCs group and non-CPCs group with respect to gender, ASA physical status, BMI, smoking habits, tumor location, nodal stage, pulmonary function test and type of surgery (*P* > 0.05). Before the surgery, the pre-existing cardiovascular medications in patients with or without CPCs did not differ significantly except beta-blocker. Preoperative comorbidities including hypertension and coronary artery disease (CAD) were more frequently observed in patients with CPCs (*P* < 0.05). The percentage of prior cardiothoracic surgery history was similar for patients with CPCs compared with those without CPCs (*P* > 0.05). Patients who suffered CPCs showed a significantly higher resting heart rate, RCRI score and clinical stage than those without CPCs (*P* < 0.05).

**Table 1:** Demographics and clinical data of patients with or without CPCs

Patient characteristics	CPCs (*n* = 42)	Non-CPCs (*n* = 138)	*P*-value
Age (year)	66.9 ± 9.5	61.3 ± 8.3	< 0.01*
Gender, n (%)			
Male	23 (54.8%)	65 (47.1%)	0.385
Female	19 (45.2%)	73 (52.9%)	–
ASA physical status, n (%)			
II	8 (19.0%)	36 (26.1%)	0.634
III	23 (54.8%)	71 (51.4%)	–
IV	11 (26.2%)	31 (22.5%)	–
BMI (kg/m^2^)	23.5 ± 4.1	23.1 ± 3.8	0.558
Current smoker, n (%)	11 (26.2%)	32 (23.2%)	0.690
Preoperative medications, n (%)			
Beta-blocker	6 (14.3%)	42 (30.4%)	0.038*
Corticosteroid	5 (11.9%)	11 (8.0%)	0.433
Calcium channel antagonist	6 (14.3%)	26 (18.8%)	0.499
ACEI or ARB	5 (11.9%)	22 (15.9%)	0.521
Diuretic	6 (14.3%)	19 (13.8%)	0.932
Anti-platelet	8 (19.0%)	33 (23.9%)	0.510
Statin	7 (16.7%)	25 (18.1%)	0.830
Resting heart rate (beats/min)	92.2 ± 8.2	82.7 ± 7.9	< 0.01*
Prior cardiothoracic surgery, n (%)	5 (11.9%)	18 (13.0%)	0.847
Comorbidities, n (%)			
Diabetes	7 (16.7%)	18 (13.0%)	0.552
Hypertension	14 (33.3%)	24 (17.4%)	0.027*
COPD	14 (33.3%)	32 (23.2%)	0.187
CAD	12 (28.6%)	20 (14.5%)	0.037*
CKD	2 (4.8%)	7 (5.1%)	0.936
Prior myocardial infarction	6 (14.3%)	15 (10.9%)	0.546
Atrial fibrillation	7 (16.7%)	19 (13.8%)	0.640
RCRI, n (%)			0.033*
I	15 (35.7%)	72 (52.2%)	–
II	12 (28.6%)	42 (30.4%)	–
III	15 (35.7%)	24 (17.4%)	–
Pathological type, n (%)			0.954
Squamous cell carcinoma	24 (57.1%)	82 (59.4%)	–
Adenocarcinoma	16 (38.1%)	49 (35.5%)	–
Mixed	2 (4.8%)	7 (5.1%)	–
Tumor location, n (%)			0.941
Upper	24 (57.1%)	83 (60.1%)	–
Middle	3 (7.1%)	9 (6.5%)	–
Lower	15 (35.7%)	46 (33.3%)	–
Clinical stage, n (%)			0.043*
I-II	35 (83.3%)	129 (93.5%)	–
III-IV	7 (16.7%)	9 (6.5%)	–
Nodal stage, n (%)			0.189
N0	32 (76.2%)	121 (87.7%)	–
N1	7 (16.7%)	12 (8.7%)	–
N2	3 (7.1%)	5 (3.6%)	–
Pulmonary function test			
Predicted FVC	92.5 ± 12.6	92.6 ± 13.1	0.965
Predicted FEV1	79.3 ± 18.9	82.0 ± 20.2	0.443
Type of surgery, n (%)			0.613
Segmentectomy	6 (14.3%)	19 (13.8%)	–
Pneumonectomy	7 (16.7%)	15 (10.9%)	–
Lobectomy	25 (59.5%)	82 (59.4%)	–
Bilobectomy	4 (9.5%)	22 (15.9%)	–
Breakdown of CPCs, n (%)			
Pleural effusion	15 (8.3%)	–	–
Arrhythmias	11 (6.1%)	–	–
Pneumonia	6 (3.3%)	–	–
Atelectasis	3 (1.7%)	–	–
Respiratory failure	2 (1.1%)	–	–
Pneumothorax	2 (1.1%)	–	–
ARDS	2 (1.1%)	–	–
Myocardial infarction	1 (0.6%)	–	–

*CPCs* cardiopulmonary complications, *ASA* American Society of Anesthesiologists, *BMI* Body Mass Index, *ACEI* angiotensin converting enzyme inhibitor, *ARB* angiotensin receptor blocker, *COPD* chronic obstructive pulmonary disease, *CAD*, coronary artery disease, *CKD* chronic kidney disease, *RCRI* revised cardiac risk index, *FVC* forced vital capacity, *FEV1* forced expiratory volume in 1 second, *ARDS* acute respiratory distress syndrome. *P*-values were calculated by Chi-square test, Fisher exact test or t test. *P value< 0.05

### Laboratory tests

As illustrated in Table 2, laboratory parameters were compared between patients with or without CPCs. Patients with CPCs exhibited higher expressions of cTnT, CRP and TNF-α (*P* < 0.05).

**Table 2:** Preoperative laboratory tests of patients with or without CPCs

Laboratory tests	CPCs (*n* = 42)	Non-CPCs (*n* = 138)	*P*-value
Hemoglobin(mg/dl)	13.1 ± 2.5	12.9 ± 3.0	0.695
Albumin (g/dL)	3.9 ± 0.4	4.0 ± 0.5	0.238
cTnT(ng/L)	12.3 ± 4.1	9.7 ± 2.8	< 0.01*
CRP (mg/L)	8.3 ± 5.3	6.1 ± 4.1	0.005*
TNF-α(nmol/L)	9.8 ± 3.5	7.7 ± 2.5	< 0.01*
Creatinine(mg/dL)	1.0 ± 0.4	0.9 ± 0.3	0.083
Urea(mmol/L)	6.5 ± 2.3	5.9 ± 2.5	0.167

*CPCs* cardiopulmonary complications, *cTnT* cardiac troponin T, *CRP* C-reactive protein, *TNF-α* tumor necrosis factor-α. *P*-values were calculated by t test or Mann–Whitney U-test. *P value< 0.05

### Risk factors for CPCs

All the potential risk factors associated with CPCs mentioned above were enrolled into the final multivariate logistic regression analysis. As shown in Table 3, elevated preoperative resting heart rate was an independent risk factor for postoperative CPCs during patients after resection for lung cancer (OR: 4.48, 95% CI: 1.17–18.42, *P* = 0.021).

**Table 3:** Preoperative risk factors associated with CPCs by multiple logistic regression analysis

Parameter	CPCs		
	OR	95% CI	*P* value
Age	0.58	0.22–1.62	0.13
Beta-blocker	0.94	0.88–1.03	0.094
Resting heart rate	4.48	1.17–18.42	0.021*
Hypertension	5.04	0.93–17.54	0.087
CAD	0.38	0.13–1.17	0.079
RCRI	0.95	0.92–1.01	0.10
Clinical stage (I/II x III/IV)	0.89	0.73–1.04	0.23
cTnT	0.92	0.81–1.08	0.31
CRP	1.02	0.88–1.18	0.84
TNF-α	1.09	0.82–1.48	0.42

*CPCs* cardiopulmonary complications, *CAD* coronary artery disease, *RCRI* revised cardiac risk index, *cTnT* cardiac troponin T, *CRP* C-reactive protein, *TNF-α* tumor necrosis factor-α. *CI*: Confidence Interval, *OR* Odds Ratio. *P value < 0.05

### Predictive role of resting heart rate for CPCs

**Fig. 1:**
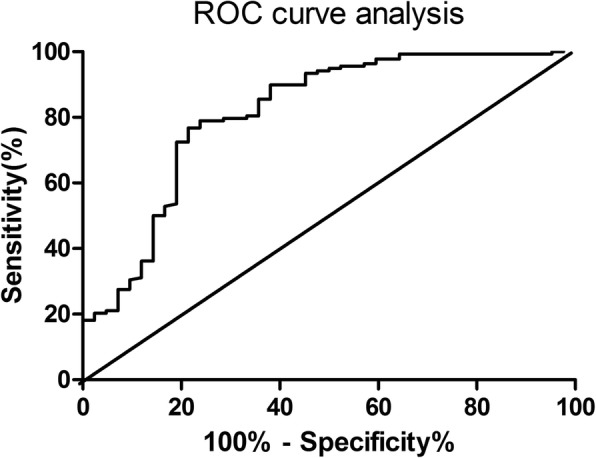
ROC curve analysis of resting heart rate for CPCs in patients after resection for lung cancer. An elevated resting heart rate was a predictor for CPCs with a cut-off value of 86 beats/min (AUC: 0.813, specificity: 80.95%, sensitivity: 72.46%, *P* < 0.001). ROC, receive operating characteristic; CPCs, cardiopulmonary complications; AUC, area under curve

The predictive role of resting heart rate for CPCs was analyzed by ROC curve analysis. An elevated resting heart rate was a predictor for CPCs with a cut-off value of 86 beats/min (AUC: 0.813, specificity: 80.95%, sensitivity: 72.46%, *P* < 0.001, see Fig. 1).

## Discussion

As the best cure strategy in patients with resectable NSCLC, surgery is still associated with a high complication rate [12]. CPCs are reported to be a major source of morbidity and mortality in the acute phase after surgery [13]. Previous studies performed in patients after lung cancer surgery have revealed that CPCs, with a high prevalence ranged 20–35%, have a profound effect on both short-term and long-term outcomes [13, 14]. The principal finding of the current single-center cohort study was that an elevated preoperative resting heart rate (cut-off value: 86 beats/min) was associated with an increased incidence of CPCs in patients after resection for lung cancer. The current study was the first to identify the possible relationship between preoperative resting heart rate and CPCs within 30 days.

Previous studies have hypothesized that a preoperative resting heart rate over 87 beats/min is significantly associated with impaired autonomic, cardiovascular function and subclinical cardiac failure [9], which is quite consistent with our results. Preoperative diagnosis of cardiac failure is closely related to increased morbidity and mortality in patients undergoing non-cardiac surgery [15]. Furthermore, a close association between increased heart failure and progressed resting heart rate has also been exposed in elderly healthy participants [16]. Previous studies have indicated that advanced age, elevated baseline heart rate, increased serum expressions of glucose, albumin and creatinine are all independent predictors of incident heart failure in elderly patients [17], which is in accordance with our results. Increasing evidence has indicated that preoperative usage of beta-blockade is associated with improved outcomes in patients at high risk of cardiac events after non-cardiac surgery [18]. Furthermore, the preoperative beta-blockade withdrawal is associated with an increased mortality and risk of postoperative adverse cardiac events in vascular surgical patients [19]. However, in contrast, another review by Giles et al. indicates that chronic medication therapy of beta-blockade correlates with an increased risk of myocardial infarction in a surgical population [20]. In contrast, our results showed no predictive value for beta blockade medication on postoperative CPCs.

It has been well-understood that elevated heart rate with stable coronary artery stenosis impairs the distribution of subendocardial and epicardial blood flow with the reason that shortened diastolic time interval leads to myocardial dysfunction and subendocardial ischemia [21, 22]. Numerous evidence is in support of the consistent association between perioperative heart rate and cardiovascular mortality in patients with high-risk cardiac events [23]. Recent studies have demonstrated the predictive value of intraoperative tachycardia for adverse outcomes including higher ICU admission, prolonged hospital stay and increased mortality in patients with major non-cardiac surgeries [24]. Heart rate has also been indicated as a significant predictive factor for overall survival during patients with septic shock [25]. Previous data have found an independent association between an increase of heart rate at 10 beats/min and an increased risk of cardiac failure at 11% [26].

Patients with higher preoperative heart rate are at increased risk of postoperative CPCs. The underlying mechanisms linking heart rate to CPCs still remain unclear, but some potential mechanisms may promote the CPCs risk in subjects after resection for lung cancer. Elevated heart rate links closely to coronary blood flow, myocardial injury and myocardial oxygen demand. The supply-demand imbalance of myocardial oxygen caused by elevated heart rate may be a possible explanation for the induction of postoperative CPCs. As a result of lower stroke volume, heart rate is subsequently increased to maintain the cardiac output. Increased CPCs risk can be ascribed to the oxygen delivery, which is supported by consistent relationship between elevated heart rate and reduced VO_2_ [27].

We conclude that patients with an elevated preoperative heart rate before lung surgery may have an increased risk of postoperative CPCs. However, why higher preoperative heart rate is associated with increased complications after lung cancer surgery remains unclear. The surgeon and anesthetist can differentiate the patients according to the preoperative heart rate and treat them accordingly. Predicting CPCs helps the surgeon to evaluate the hospital stay, intensive care unit stay, economic costs, and outcomes. Furthermore, the surgeon can decide surgery plans as well as inoperability after physiological assessment. We recommend that those patients with preoperative heart rate (over 86 beats/min) can be categorized into high-risk surgical patients and individualized approaches to care are recommended.

We acknowledge that the study has the following study limitations. The sample size of this study was relatively small and involved mechanisms remained unclear. A long follow up and monitoring the incidence of CPCs would improve the precision and have better effects. Furthermore, we have to acknowledge that there were some residual and unmeasured confounding factors. At last, only the patients of certain disease (lung cancer) were enrolled, whether preoperative heart rate could be a more general predictor for prognosis than specifically for certain diseases remained unknown.

## Conclusions

In conclusion, our results revealed that elevated preoperative resting heart rate was associated with an increased risk of postoperative CPCs in patients after resection for lung cancer.

## Abbreviations

ACEI: Angiotensin converting enzyme inhibitor
ARB: Angiotensin receptor blocker
ASA: American Society of Anesthesiologists
BMI: Body Mass Index
CAD: Coronary artery disease
CI: Confidence Interval
CKD: Chronic kidney disease
COPD: Chronic obstructive pulmonary disease
CPCs: Cardiopulmonary complications
CRP: C-reactive protein
cTnT: Cardiac troponin T
ELISA: Enzyme-linked immunesorbent assays
FEV1: Forced expiratory volume in 1second
FVC: Forced vital capacity
OR: Odds Ratio
RCRI: Revised cardiac risk index
ROC: Receiver operating characteristic
SD: Standard error
TNF-α: Tumor necrosis factor-α
